# Effects of a neurokinin-1 receptor antagonist in the acute phase after thoracic spinal cord injury in a rat model

**DOI:** 10.3389/fnmol.2023.1128545

**Published:** 2023-05-12

**Authors:** Guoli Zheng, Anna-Kathrin Harms, Mohamed Tail, Hao Zhang, Alan Nimmo, Thomas Skutella, Karl Kiening, Andreas Unterberg, Klaus Zweckberger, Alexander Younsi

**Affiliations:** ^1^Department of Neurosurgery, Heidelberg University Hospital, Heidelberg, Germany; ^2^College of Medicine and Dentistry, James Cook University, Cairns, QLD, Australia; ^3^Department of Neuroanatomy, Institute for Anatomy and Cell Biology, University of Heidelberg, Heidelberg, Germany

**Keywords:** spinal cord injury, animal model, neurokinin-1 (NK1) antagonist, blood spinal cord barrier, inflammation, behavioral assessment

## Abstract

**Objective:**

Disruption of the blood-spinal cord barrier (BSCB) with subsequent edema formation and further neuroinflammation contributes to aggravation of spinal cord injury (SCI). We aimed to observe the effect of antagonizing the binding of the neuropeptide Substance-P (SP) to its neurokinin-1 (NK1) receptor in a rodent SCI model.

**Methods:**

Female Wistar rats were subjected to a T9 laminectomy with or without (Sham) a T9 clip-contusion/compression SCI, followed by the implantation of an osmotic pump for the continuous, seven-day-long infusion of a NK1 receptor antagonist (NRA) or saline (vehicle) into the intrathecal space. The animals were assessed *via* MRI, and behavioral tests were performed during the experiment. 7 days after SCI, wet & dry weight and immunohistological analyses were conducted.

**Results:**

Substance-P inhibition *via* NRA showed limited effects on reducing edema. However, the invasion of T-lymphocytes and the number of apoptotic cells were significantly reduced with the NRA treatment. Moreover, a trend of reduced fibrinogen leakage, endothelial and microglial activation, CS-GAG deposition, and astrogliosis was found. Nevertheless, only insignificant general locomotion recovery could be observed in the BBB open field score and the Gridwalk test. In contrast, the CatWalk gait analysis showed an early onset of recovery in several parameters.

**Conclusion:**

Intrathecal administration of NRA might reinforce the integrity of the BSCB in the acute phase after SCI, potentially attenuating aspects of neurogenic inflammation, reducing edema formation, and improving functional recovery.

## 1. Introduction

Traumatic spinal cord injury (SCI), most commonly originating from burst/compression fractures or dislocation of the vertebral column due to traffic accidents or falls (van den Berg et al., 2010; Mothe and Tator, 2013; Lee et al., 2014), remains a disastrous event for affected patients. Unfortunately, the injury is primarily irreversible, spontaneous recovery is limited, and besides surgical decompression within 24 h and the administration of methylprednisolone within 8 h of acute SCI, no current treatment approach showed significant effects on functional recovery (Ramer et al., 2014; Fehlings et al., 2017; Badhiwala et al., 2021).

This devastating injury can be divided into a primary and a secondary phase of pathological changes: Physical forces such as compression, contusion, or laceration cause primary injury to the neuronal tissue, leading to shearing and damaging axons and blood vessels (Ackery et al., 2004). As a consequence, a cascade of secondary injury processes is initiated, including the disruption of the blood-spinal-cord-barrier (BSCB), edema formation, excitotoxicity, and inflammation, all featured more in the acute stage (within 48 h after the injury; Shende and Subedi, 2017). When the injury develops into a subacute stage (from 2 days to 2 weeks), those pathological processes further deteriorate and cause enriched glial scar and cyst formation, resulting in a physical barrier for axonal regrowth and neuronal rewiring (Plemel et al., 2014; Nagoshi and Okano, 2018).

Especially the inflammatory response to SCI has multiple facets, including microglial activation, astrocytic proliferation, leukocyte recruitment and infiltration, and the secretion of cytokines, chemokines, and neuropeptides (Ziebell and Morganti-Kossmann, 2010). Such neuropeptides include substance P (SP), neurokinin A and B, and calcitonin gene-related peptides (Zieglgänsberger, 2019). SP, an 11-amino acid long neuropeptide, is regarded as the most potent initiator of inflammation due to its role in vascular permeability and subsequent plasma protein extravasation (Holzer, 1998). The downstream effects of SP are triggered by three different tachykinin neurokinin receptors (NKRs), among which the Neurokinin-1 receptor (NK1R) plays a predominant role (Mantyh, 2002). NK1R is located in smooth muscle cells, endothelial cells, fibroblasts, and various circulating immune and inflammatory cells, but also in neurons and glial cells.

After injury to the CNS, it is immediately released and binds to the NK1R, leading to even more SP release (Nimmo et al., 2004; Leonard et al., 2013). Moreover, rapid endocytosis and internalization of the receptor after binding of SP trigger the phosphoinositide metabolism and the formation of cAMP (O’Connor et al., 2004). In a traumatic brain injury (TBI) model, SP has been associated with several aspects of inflammation, including blood–brain–barrier (BBB) disruption, circulating cell chemotaxis and adhesion, and cytokine secretion (Corrigan et al., 2016). Furthermore, on binding to the NK1R, SP has been related to transforming resident forebrain astrocytes into a reactive status after an ischemic injury (Lin, 1995). Conversely, antagonizing NK1R *via* an NK1 receptor antagonist (NRA) not only reduced the SP activity but also inhibited neurogenic inflammation and improved functional recovery in several preclinical TBI studies (Donkin et al., 2011; Corrigan et al., 2012; Ameliorate et al., 2017).

In the spinal cord of rats, a high density of SP has been discovered in dorsal root ganglion neurons (primary sensory neurons) and the dorsal horn rather than the ventral horn (Hökfelt et al., 2001; Zieglgänsberger, 2019). Similarly, a high SP immunoreactivity was found in the dorsal horn region of human spinal cords following SCI, whereas NK1R immunocreactivity showed a perivascular prevalence (Leonard et al., 2014a). However, the number of preclinical studies on the effect of NRA on secondary injury processes after SCI is limited (Zhang et al., 2008; Leonard et al., 2014b). Most of such reports have used the non-lipid-soluble antagonist n-acetyl L-tryptophan. This drug has recently been found to be inferior to a lipid-soluble and thus cell-permeable NK1R antagonist in, e.g., attenuating tau phosphorylation and improving neurological outcomes after different experimental TBI models (Donkin et al., 2011; Corrigan et al., 2021).

Therefore, our current experiment aimed to investigate how the intrathecal administration of lipid-soluble NRA modulates spinal cord edema formation, BSCB disruption, neuroinflammation and functional recovery after severe compression/contusion SCI in the acute and subacute stages.

## 2. Methods

### 2.1. Animals, experimental groups, and study design

A total of 36 female Wistar rats (200 g; Janvier Labs, Le Genest-Saint-Isle, France) were randomly assigned to three groups: Group 1 (Sham; *n* = 12), group 2 (NRA; *n* = 12) and group 3 (Vehicle; *n* = 12). During the study, three animals were housed in one cage with a 12:12 h light/dark cycle at 26°C and with food and water *ad libitum*. All surgeries and outcome assessments were blinded, and all experimental protocols were approved by the Animal Care Committee of the federal government of Baden Württemberg, Germany (ethic approval code G-285/19). After sacrifice, animals within each group were either subjected to immunohistochemistry (IHC) staining (*n* = 6) or wet/dry weight analysis (*n* = 6; Table 1).

**Table 1:** Group design and timeline of the study.

	Group 1 (Sham; *n* = 12)	Group 2 (NRA; *n* = 12)	Group 3 (Vehicle; *n* = 12)
Day −3	Acclimation to the environment and the behavioral tests		
Day −1	Baseline for all behavioral tests		
Day 0	T9 laminectomy only	T9 SCI + osmotic pump implantation for i.t. NRA-delivery	T9 SCI + osmotic pump implantation for i.t. saline-delivery
Day 1	BBB open field score only; MRI (*n* = 3/group)		
Day 3	BBB open field score only; MRI (*n* = 3/group)		
Day 7	All behavioral tests; MRI (*n* = 3/group) + sacrifice without perfusion for wet and dry weight analysis (*n* = 6/group); Sacrifice with perfusion for immunhistochemistry staining (*n* = 6/group)		

### 2.2. Surgical procedures

For all surgical procedures, animals were anesthetized with 1.5% isoflurane and a 1:1 mixture of O_2_ and N_2_O. For animals in group 2 and 3, A 28-g modified aneurysm clip (Fehlings Laboratory, Canada) was used to induce a contusion and compression SCI at the T9 level, while animals in group 1 only underwent a T9 laminectomy as sham surgery. After the T9 SCI, a skip-level laminectomy of T11 was performed, and an intrathecal microcatheter (Alzet, United States), connected to a subcutaneous osmotic micropump (1007D; Alzet, United States) at a flow speed of 0.5 μl/h, was placed subdurally with its open end over the lesion on T9, as described previously (Zhang et al., 2021). The pumps were filled with either 200 μl of the selective NRA “EUC-001” ((N-(3,5-bis-trifluoromethylbenzyl)-N-methyl-6-(4-methyl-piperazin-1-yl)-4-o-tolyl-nicotinamide); 87.5 mg/ml; a kind gift from Prof. Alan Nimmo of James Cook University (Hoffmann et al., 2005) or 0.9% NaCl and they were preloaded at room temperature for 2 h. Assignment of the pumps to the animals then took place randomly during the surgery. As a result, animals in group 2 were administered 10 mg/kg/d of EUC-001 for 7 days, with the intrathecal dosing being guided by its previous systemic use (10 mg/kg i.v.) in similar models, which is a common extrapolation given the potential for systemic distribution as well as topical action (Bernards, 2004; Hoffmann et al., 2005).

After the surgery, analgesics (0.05 mg/kg buprenorphine s.c.; Bayer, Germany, and 2 mg/kg meloxicam s.c.; Boehringer-Ingelheim, Germany) were administered for 5 days, and antibiotic prophylaxis (4 mg/kg moxifloxacin p.o.; Alcon, United States) was given for 7 days. If necessary, bladders were manually voided twice a day.

### 2.3. Behavioral tests

The Basso-Beattie-Bresnahan (BBB) open field score was used to evaluate the general hindlimb locomotion recovery. Hindlimb movement, joint movement, stepping, coordination, trunk, and tail position, as well as weight support of rats, were evaluated on a 4-min-long video using a rating scale with 0–21 points (Basso et al., 1995). The videos were taken by two cameras at different angles to ensure a whole view of animal hindlimbs during moving. Besides the baseline test, the BBB open field score was tested on days 1, 3, and 7 after SCI/sham surgery. The videos were reviewed by two blinded observers, and scores were averaged between them.

The Gridwalk test was performed to assess fine sensory-motor coordination. Animals needed to run through a 1-m-long pathway of randomly placed metal grids. Misplacement of a hindlimb between the bars was defined as a stepping error and stepping errors were counted and summed up over four runs by two blinded observers (Metz et al., 2000). The test took place as an endpoint of the experiment 7 days after SCI/sham surgery and both observers’ results were averaged.

The CatWalk XT^®^ automated gait analysis was applied to objectively quantify several locomotion characteristics as an endpoint of the experiment 7 days after SCI/sham surgery as well. To this end, animals ran voluntarily over a horizontal glass walkway, illuminated by a green LED light to highlight areas where the paws touch the glass, and were filmed by a high-speed color camera placed below. At least three compliant and uninterrupted runs were recorded for one trial and then further processed by the CatWalk XT^®^ software (version 10.5; Noldus Information Technology, Netherlands; Hamers et al., 2006; Zheng et al., 2021). A maximum run variation of 60%, a camera gain of 16.99 decibels (dB), and a detection threshold of 0.1 arbitrary units (a.u.) were set as default detection conditions for all runs. Every run was automatically classified by the software and then manually reviewed to remove the misclassification such as nose or the abdomen contacts with the walkway or the mislabeling of the paws by a blinded observer.

### 2.4. Magnetic resonance imaging and wet and dry weight

To determine the severity of spinal cord edema, MRI was performed on three randomly selected animals on day 1 post-SCI and on the same animals on days 3, and 7 post-SCI. T2-weighted images with a T2_RARE_Nav_axial sequence (TE = 85 ms, TR = 3386.68 ms, slice thickness 2 mm, mean values = 8, slices 15, flip angle 180°, voxel size 0.3125 * 0.3125 * 2 mm^3^, matrix size 192 * 192) and a T2_RARE_Nav_sagittal sequence (TE = 72 ms, TR = 1,920.17 ms, slice thickness 2 mm, mean values = 8, slices 10, flip angle 180°, voxel size 0.3125 * 0.3125 * 2 mm^3^, matrix size 192 * 192) were acquired by a Bruker ICon 1 T scanner (Ettlingen, Germany; Freitag et al., 2015). The images were then evaluated with the software ImageJ (National Institute of Health, Bethesda, United States). Every spinal cord was divided into axial sections from 4 mm rostral to 4 mm caudal to the lesion epicenter at the T9 level. As a surrogate for tissue water content, the grayscale of the spinal cord was measured on each axial section by placing a region of interest (ROI) around the spinal cord contours, averaged, and compared between the groups at the respective time points.

For the same purpose, the six animals from each group that had received MR imaging were sacrificed for a wet & dry weight analysis at the end of the experiment 7 days after SCI/sham surgery. To this end, after euthanasia with 5% isoflurane, 10-mm-long pieces were extracted from the cervical (centered at the C5-6 level, Cv) and the thoracic (centered at the T9 level, Th) spinal cord. The spinal cord pieces were weighed (wet weight, WW) before being put into a preheated 100°C oven for 48 h. The dried spinal cord pieces were then weighed again (dry weight, DW). To improve the accuracy of this assessment and to diminish the bias caused by individual size/volume differences, the corrected value of the amount of tissue water, as a surrogate for spinal cord edema, was calculated by the following formula:


$$\mathrm{Corrected water amount}=\frac{\left(\mathrm{WW}\;\mathrm{of}\;\mathrm{Th}-\mathrm{DW}\;\mathrm{of}\;\mathrm{Th}\right)}{\left(\mathrm{WW}\;\mathrm{of}\;\mathrm{Cv}-\mathrm{DW}\;\mathrm{of}\;\mathrm{Cv}\right)}.$$

### 2.5. Immunohistochemistry staining

At the end of the experiment 7 days after SCI/sham surgery, the other six animals from each group were also sacrificed by euthanasia with 5% isoflurane but then perfused with 50 ml 0.1 M cold phosphate-buffered saline (PBS) followed by 150 ml 4% paraformaldehyde (PFA). The spinal cords were then resected, and after post-fixation in 4% PFA for 24 h and cryoprotection in 30% sucrose for 48 h, pieces with a length of 10 mm, centered at the lesion epicenter, were cut, and embedded in tissue embedding medium (Sakura Finetek Europa B.V., Alphen aan den Rijn, Netherlands). A cryostat (Leica Biosystems, Nussloch, Germany) was used to further cut those pieces into 30 μm thick cross-sections which were then dried and stored at −80°C until further processing.

For immunohistochemistry staining, the spinal cord sections were first blocked with a blocking solution (5% non-fat milk powder, 1% bovine serum albumin, and 0.3% Triton-X100) for 1 h and then incubated with the following primary antibodies overnight: Anti-substance P (1:200; sc-21,715, Santa Cruz, United States), anti-NK-1R (1:200; sc-365,091, Santa Cruz, United States), anti-Fibrinogen (1:500; sc-398,806, Santa Cruz, United States), anti-CD31 (1:100; MA1-80069, Invitrogen, United States), anti-CD3 (1:200; MCA772, Bio-Rad, Germany), anti-Iba1 (1:200; NB100-1028, Novus Biologicals, United States), anti-TMEM119 (1:200; ab185337, Abcam, United States), anti-Arginase1 (1:200, PA5-29645, Invitrogen, United States), anti-caspase 3 (1:400; #9661, Cell signaling, United States), anti-CSPG (1:400; MAB1581, Millipore, United States), and anti-GFAP (1:250; Abcam, United States). The spinal cord sections were then incubated with secondary antibodies (1:500; ab150129, ab175700, ab150075, Abcam, United States) and DAPI (1:10000; #6335.1, Carl Roth, Germany) for 1 h and sealed with mounting medium before imaging analysis.

### 2.6. Imaging analysis

All immunofluorescence staining images were captured using a confocal laser microscope (LSM 700; Carl-Zeiss, Germany) with the ZEN microscope software (ZEN 2010; Carl Zeiss, Germany). Depending on the secondary antibodies, the following wavelengths were used: 488, 568, and 647 nm (Alexa Fluor) or 405 nm (DAPI).

For quantitative assessment of the Substance P release, BSCB-integrity, chondroitin sulfate glycosaminoglycan (CS-GAG) deposition, and astrogliosis, the immunointensity of the staining of Fibrinogen, CSPG, or GFAP was measured on 11 different spinal cord cross-sectional images (0 μm, ±  240 μm, ±  480 μm, ± 720 μm, ± 960 μm and ± 1,200 μm from the lesion epicenter) by ImageJ (National Institute of Health; Bethesda, United States). The results were averaged for each animal and then compared between groups.

To quantify the number of NK-1R expressing cells, apoptotic cells, vascular endothelial adhesions, infiltrating T-lymphocytes, and activated microglia, a semi-automatic cell-counting algorithm for ImageJ was used on 11 spinal cord cross-sectional images per animal, which had the exact distinct distances to the lesion epicenter (0 μm, ± 240 μm, ± 480 μm, ± 720 μm, ± 960 μm and ± 1,200 μm), as described previously (Younsi et al., 2020). The number of positively stained cells was divided by the ROI area and was then presented as the cell density (cells/mm^2^). Counting results were averaged for each animal and then compared between groups.

### 2.7. Statistical analysis

All results are given as mean ± standard error of the mean (SEM). For the statistical comparison of the BBB open field score, the Gridwalk test, and the CatWalk XT gait analysis results between groups and time points, a two-way repeated measure analysis of variance (ANOVA), followed by a Tukey test for multiple comparisons, was used. Means between multiple groups in the other assessments were analyzed using one-way repeated measures ANOVAs followed by post-hoc Tukey-HSD-tests. Normality assumption was confirmed prior to all parametric analyses using Shapiro–Wilk normality tests, and a value of p of *p* < 0.05 was considered significant. All statistical analyses were performed using the Prism 9.0 (GraphPad Software, San Diego, CA, United States).

## 3. Results

### 3.1. SP and NK-1R expression

**Figure 1:**
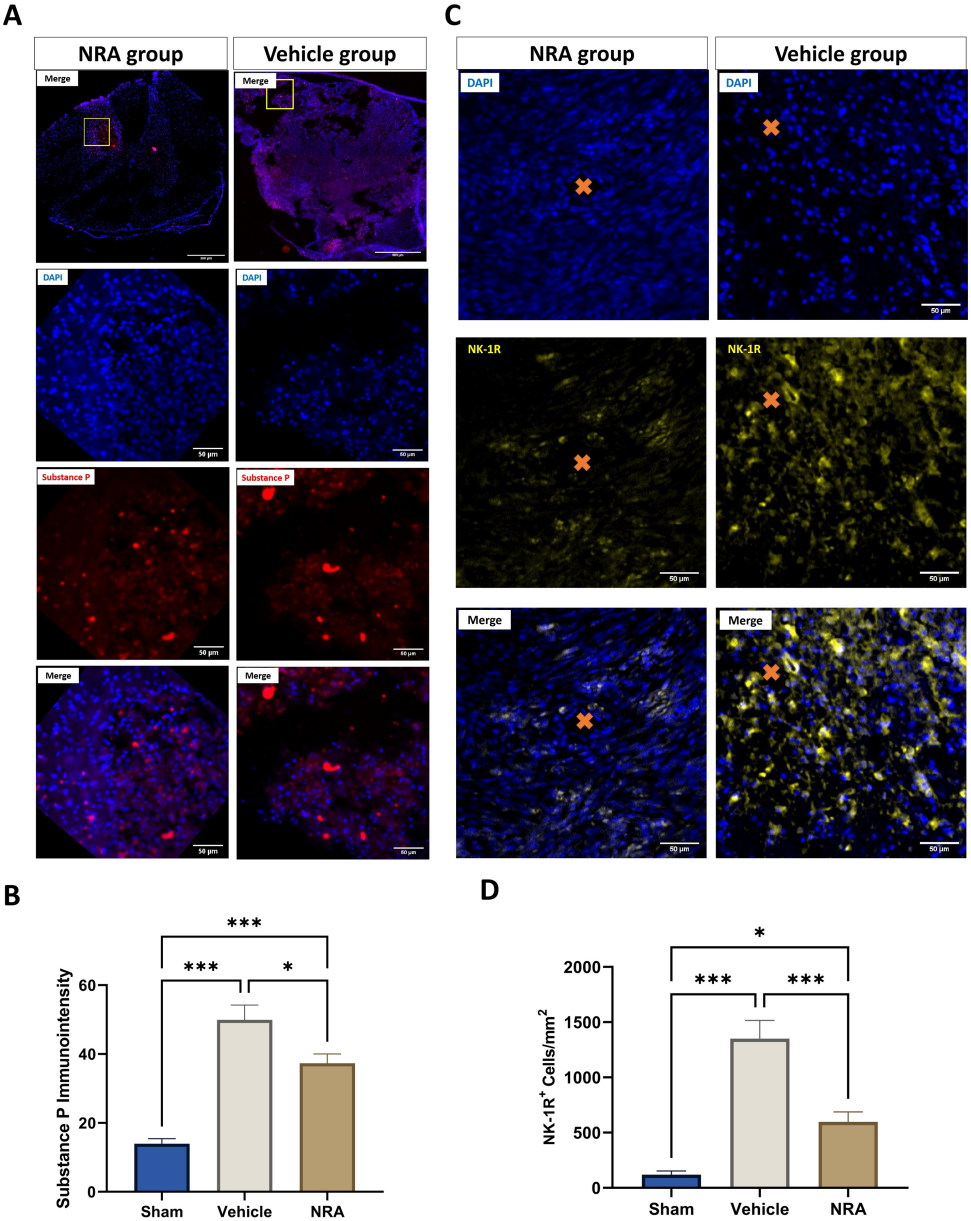
Spinal cord cross-sections of one NK1 receptor antagonist (NRA) and one vehicle animal at 10 × and 40 × magnification stained for SP (**A**, red) and NK-1R (**B**, yellow). **(C)** Mean immunointensity of SP in the spinal cord 7 days after SCI/sham surgery. **(D)** Mean cell density (cells/mm^2^) of DAPI^+^&NK-1R^+^ cells in the spinal cord 7 days after SCI/sham surgery (Orange cross, microvessel in the spinal cord) (*n* = 6/group; one-way repeated measures ANOVA followed by *post-hoc* Tukey-HSD-test; ^***^p < 0.001; ^*^p < 0.05).

To verify the antagonizing effect of the 7-day-long i.t. NRA-administration, we quantified the expression of SP and NK-1R within the spinal cord 7 days after SCI/sham surgery (Figures 1A–D).

We found that the SP immunointensity in both, the NRA (37.3 ± 2.7) and vehicle group (49.9 ± 4.3), was significantly higher than in the uninjured Sham group (14 ± 1.4, both *p* < 0.001), indicating an upregulation of SP after the injury. Of note, the SP expression was mainly located in the dorsal horn or around the lesion site in the Sham or NRA animals, whereas it was more diffuse in Vehicle animals (Figure 1A 10 × magnification).

However, a significant decrease in SP immunointensity was discovered in the NRA group compared to the vehicle group (*p* = 0.03). Moreover, we noticed significantly less NK-1R-expressing cells in the NRA group compared to the vehicle group (598 ± 90 cells/mm^2^ vs. 1,351 ± 166 cells/mm^2^, *p* < 0.001). Nevertheless, NK-1R expression was still significantly higher in the NRA group than in the Sham group (119 ± 33 cells/mm^2^, *p* < 0.05), indicating a partial but effective blockade of the neurogenic inflammation *via* a neurokinin-involved pathway.

### 3.2. Spinal cord edema

**Figure 2:**
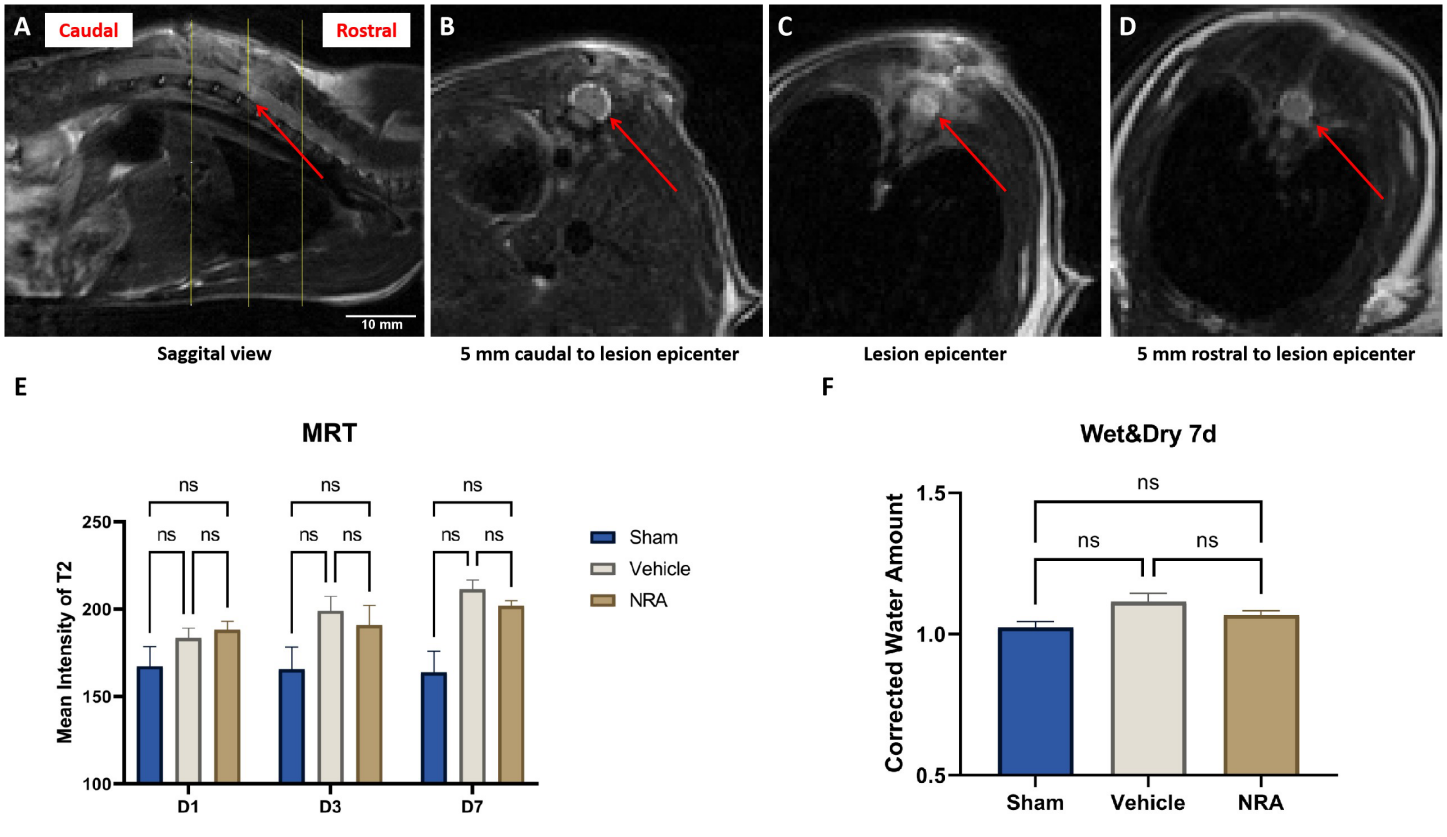
**(A)** Sagittal view of a T2-weighted MRI of a vehicle animals’ spinal cord 7 days after SCI. The red arrow indicates the epicenter of the spinal cord lesion at T9, and the yellow lines indicate three representative axials views **(A)**. **(B–D)** Axial views of the vehicle animals’ T2-weighted MRI according to the yellow lines in **(A)**. The red arrows point at the spinal cord, surrounded by the cerebrospinal fluid. **(E)** Mean signal intensity of the spinal cords on T2-weighted MRI in the Sham, NRA, and vehicle group 1, 3, and 7 days after SCI/sham surgery (*n* = 3/group; one-way repeated measures ANOVAs followed by *post-hoc* Tukey-HSD-tests; ^*^p < 0.05; ns = not significant). **(F)** Corrected water loss (%) in the thoracic spinal cord of the Sham, NRA, and vehicle animals 7 days after SCI/sham surgery (*n* = 6/group; one-way repeated measures ANOVA followed by *post-hoc* Tukey-HSD-test; ns = not significant).

Since T2-weighted MRI sequences can reflect the level of tissue water, we measured the signal intensity of the spinal cord (Figures 2A–D) as a surrogate for the severity of edema at days 1, 3 and 7 after SCI/sham surgery.

We observed that the spinal cord signal intensity and thus edema generally tended to increase in the injured animals of the NRA and the vehicle group from day 1 to 7 post-SCI (Figure 2E). In addition, at the end of the experiment, spinal cord edema in the vehicle group was significantly higher than in the Sham group (211.3 ± 5.5 vs. 163.9 ± 12.1; *p* = 0.01). Although the NRA-treated animals showed less spinal cord edema than the untreated vehicle animals at d3 and d7 post-SCI, the intergroup difference did not reach statistical significance (D3: 190.9 ± 11.2 vs. 199.0 ± 8.3; *p* = 0.84, D7: 201.8 ± 3.0 vs. 211.3 ± 5.5; *p* = 0.28).

As a more direct surrogate of spinal cord edema, we used the wet and dry weight method to quantify the corrected amount of tissue water in the freshly extracted thoracic spinal cord 7 days after SCI/sham surgery. The average corrected water amount in the Sham group was 1.01 ± 0.01 (Figure 2F), indicating a consistency of the water amount in the cervical and thoracic spinal cord. Compared to the Sham group, the corrected water amount of the NRA group and vehicle group was slightly higher (1.07 ± 0.02 and 1.12 ± 0.03), without reaching a statistically significant difference (*p* = 0.54 and *p* = 0.09). Similarly, no significant difference in water amount and thus spinal cord edema could be observed 7 days after SCI between animals in the NRA and the vehicle group (*p* = 0.35).

### 3.3. Blood-spinal cord barrier integrity

**Figure 3:**
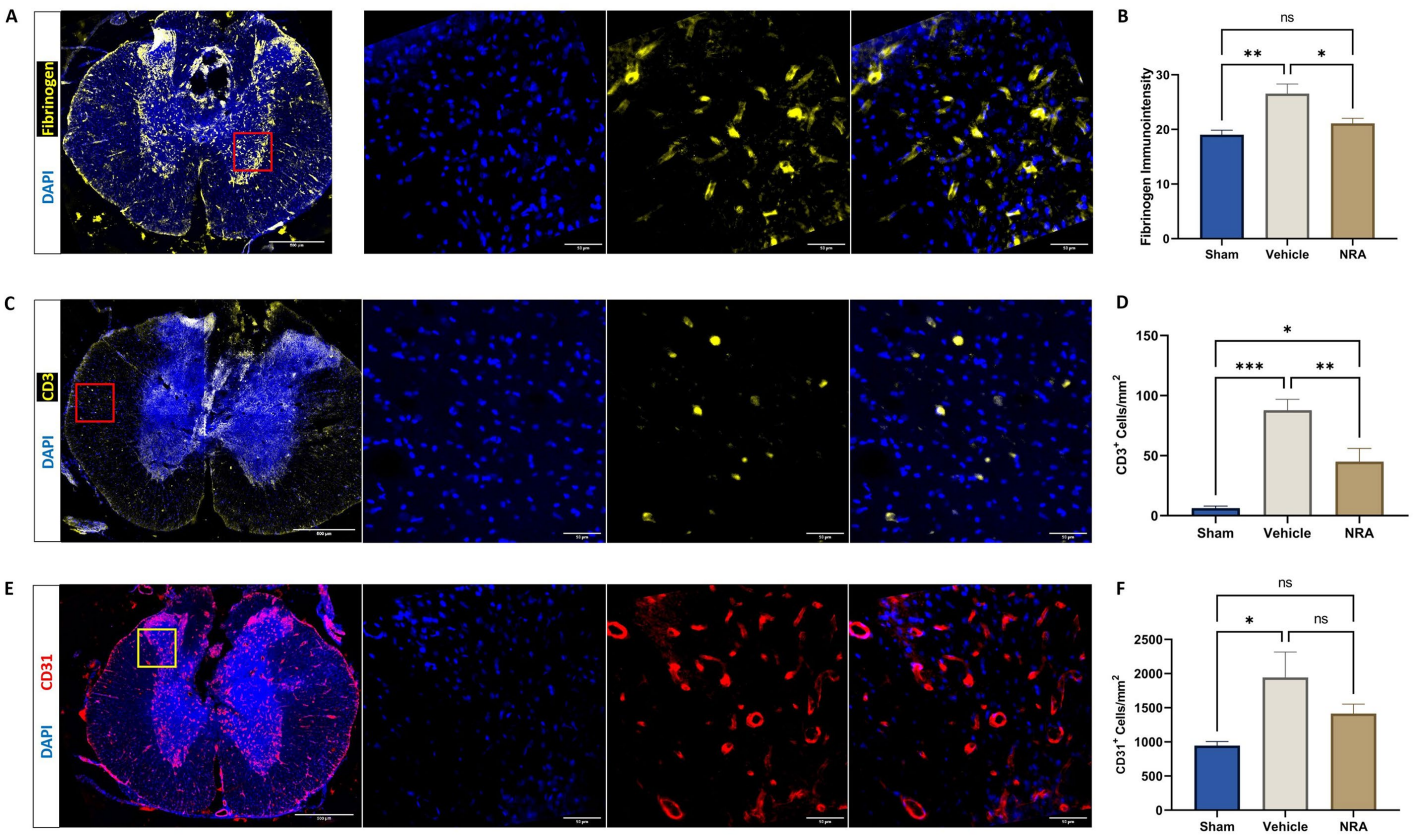
Spinal cord cross-sections of a vehicle animal at 10 × and 40 × magnification stained for Fibrinogen (**A**, yellow), CD3 (**C**, yellow), and CD31 (**E**, red). **(B)** Mean immunointensity of Fibrinogen in the spinal cord 7 days after injury. **(D,F)** Mean cell density (cells/mm^2^) of DAPI^+^&CD3^+^ T-lymphocytes **(D)** and DAPI^+^&CD31^+^ endothelial cells **(F)** in the spinal cord 7 days after SCI/sham surgery (*n* = 6/group; one-way repeated measures ANOVA followed by *post-hoc* Tukey-HSD-test; ^***^p < 0.001; ^**^p < 0.01; ^*^p < 0.05; ns = not significant).

While the average immunointensity of Fibrinogen in the spinal cord of uninjured Sham animals was 19.0 ± 0.84, its immunointensity and thus its leakage over the BSCB was significantly higher in injured vehicle animals (26.6 ± 1.66; *p* = 0.002). However, no such significant difference could be observed between Sham animals and injured animals in the NRA group (21.1 ± 0.93; *p* = 0.50). Nevertheless, the NRA-treatment significantly changed the Fibrinogen-immunointensity compared to vehicle animals 7 days after SCI (*p* = 0.02; Figures 3A,B).

The density of CD3^+^&DAPI^+^ T-lymphocytes in the spinal cord was found to be significantly higher in the vehicle group compared to the Sham group (87.9 ± 9.04 CD3^+^&DAPI^+^ cells/mm^2^ vs. 6.2 ± 1.9 CD3^+^&DAPI^+^ cells/mm^2^; *p* < 0.001), indicating a more pronounced infiltration of those immune cells over the BSCB. While animals in the NRA group still showed a significantly higher density of T-lymphocytes compared to Sham animals (45.0 ± 11.0 CD3^+^&DAPI^+^ cells/mm^2^ vs. 6.2 ± 1.9 CD3^+^&DAPI^+^ cells/mm^2^; *p* = 0.02), the NRA-treatment had notably reduced the infiltration of T-lymphocytes and thus the disruption of the BSCB compared to untreated vehicle animals 7 days after the injury (*p* = 0.009; Figures 3C,D).

Moreover, the density of CD31^+^&DAPI^+^ endothelial cells was significantly increased in injured vehicle animals compared to uninjured Sham animals (1,946 ± 369 CD31^+^&DAPI^+^ cells/mm^2^ vs. 947 ± 59 CD31^+^&DAPI^+^ cells/mm^2^; *p* = 0.03), indicating enhanced leukocyte adhesion and migration over the BSCB. However, the difference between NRA-treated animals and Sham animals remained insignificant (1,415 ± 139 CD31^+^&DAPI^+^ cells/mm^2^ vs. 947 ± 59 CD31^+^&DAPI^+^ cells/mm^2^; *p* = 0.29). Nevertheless, no effect of the 7-day-long NRA-treatment on the density of CD31^+^&DAPI^+^ endothelial cells compared to vehicle animals could be observed (*p* = 0.29; Figures 3E,F).

### 3.4. Microglial activation

**Figure 4:**
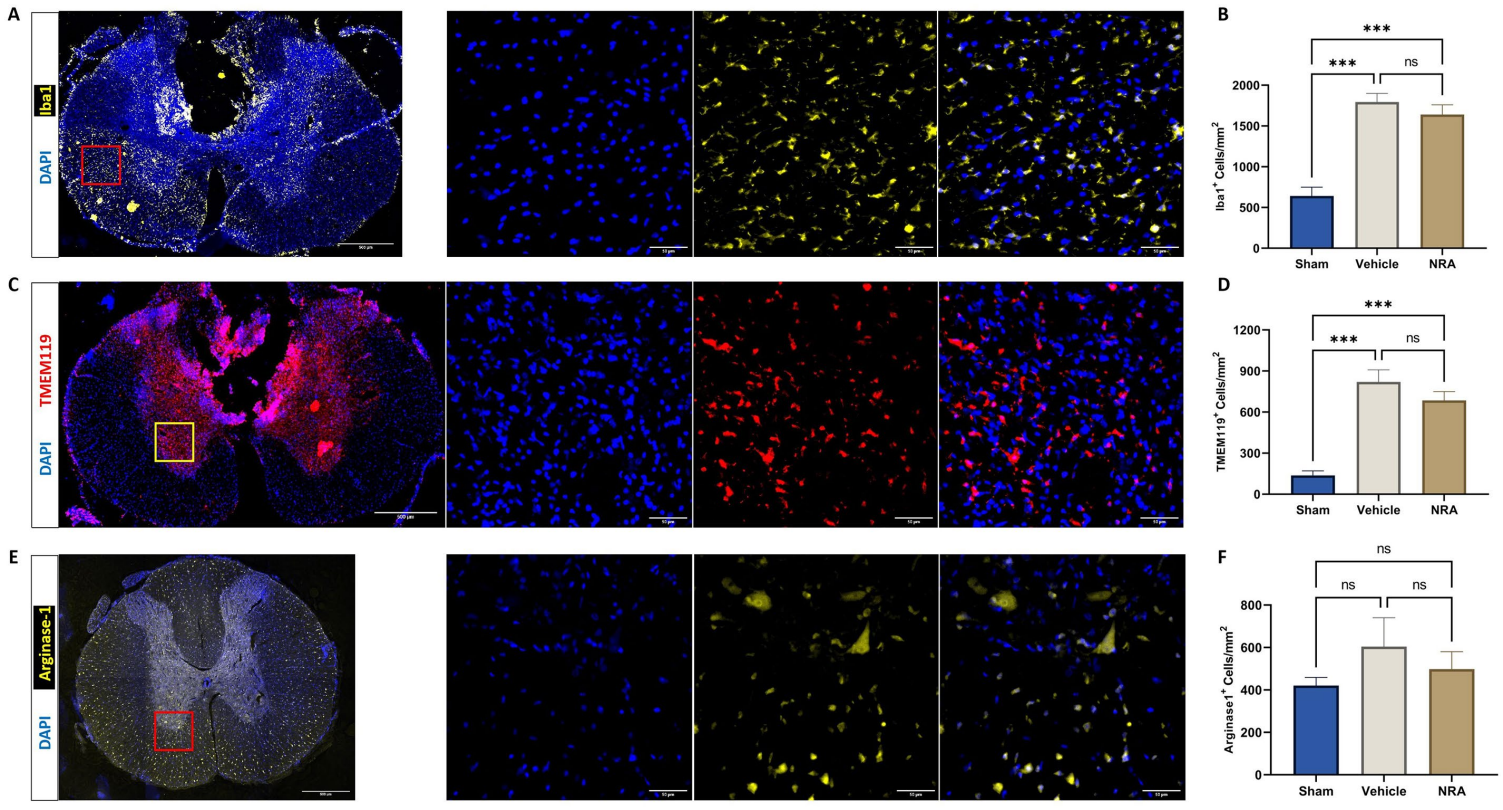
Spinal cord cross-sections of a vehicle animal at 10 × and 40 × magnification stained for Iba1 (**A**, yellow), TMEM119 (**C**, red), and Arg1 (**E**, yellow). **(B,D,F)** Mean cell density (cells/mm^2^) of DAPI^+^&Iba1^+^ infiltrating macrophages **(B)**, DAPI^+^&TMEM119^+^ resident microglia **(D)**, and DAPI^+^&Arg1^+^ M2-polarized microglia **(F)** in the spinal cord 7 days after SCI/sham surgery (*n* = 6/group; one-way repeated measures ANOVA followed by *post-hoc* Tukey-HSD-test; ^***^p < 0.001; ns = not significant).

Compared to the uninjured Sham group (642 ± 107 DAPI^+^&Iba1^+^ cells/mm^2^), the thoracic SCI resulted in a significant increase of Iba1-expressing infiltrating macrophages in the NRA (1,641 ± 118 DAPI^+^&Iba1^+^ cells/mm^2^; *p* < 0.001) and the vehicle animals (1,794 ± 105 DAPI^+^&Iba1^+^ cells/mm^2^; *p* < 0.001; Figures 4A,B). However, the difference between NRA-treated animals and vehicle animals remained insignificant (*p* = 0.59). Similarly, a significant increase of TMEM119-expressing resident microglia in the NRA group (685 ± 65 DAPI^+^&TMEM119^+^ cells/mm^2^) and the vehicle group (820 ± 88 DAPI^+^&TMEM119^+^ cells/mm^2^) could be observed when compared to the Sham group (138 ± 32 cells/mm^2^; *p* < 0.001 respectively; Figures 4C,D) and there was no significant difference between the NRA animals and the vehicle animals in terms of resident microglia as well (*p* = 0.36).

We could further observe a slightly increased density of Arginase 1 expressing M2-polarized microglia in the spinal cord of injured NRA (499 ± 82 DAPI^+^&Arg1^+^ cells/mm^2^) compared to uninjured Sham animals (421 ± 38 DAPI^+^&Arg1^+^ cells/mm^2^), which did not reach statistical significance (*p* = 0.85). However, the highest density of M2-polarized microglia with potentially anti-inflammatory effects was found in the vehicle animals (604 ± 136 DAPI^+^&Arg1^+^ cells/mm^2^), without a significant difference to the NRA (*p* = 0.72) and the Sham animals (*p* = 0.43; Figures 4E,F).

### 3.5. Apoptosis, CS-GAG deposition, and astrogliosis

**Figure 5:**
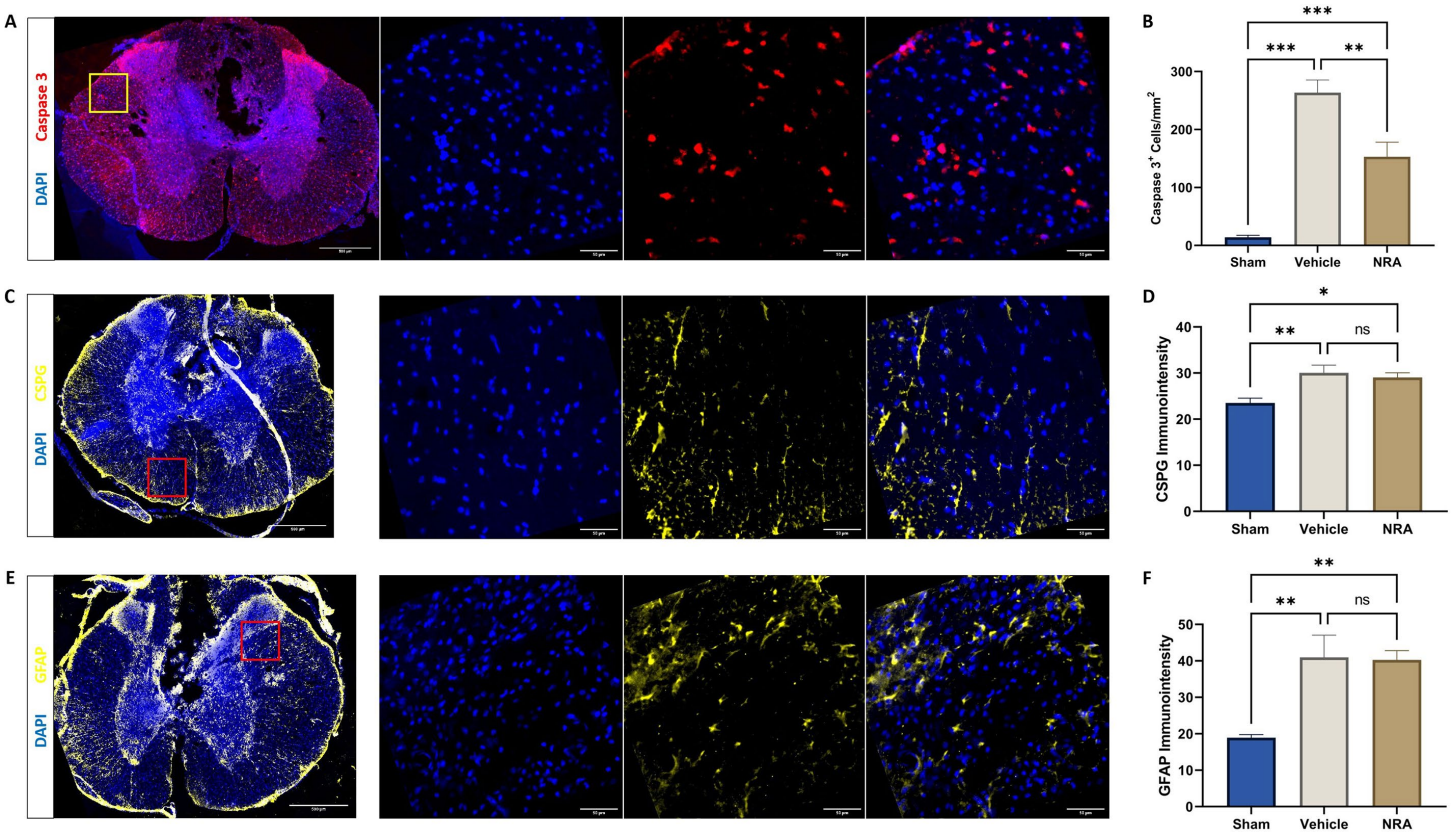
Spinal cord cross-sections of a vehicle animal at 10 × and 40 × magnification stained for Caspase 3 (**A**, red), CSPG (**C**, yellow), and GFAP (**E**, yellow). **(B)** Mean cell density (cells/mm^2^) of DAPI^+^&Caspase 3^+^ apoptotic cells and mean immunointensity of CSPG **(D)** and GFAP **(F)** in the spinal cord 7 days after SCI/sham surgery (*n* = 6/group; one-way repeated measures ANOVA followed by *post-hoc* Tukey-HSD-test; ^***^p < 0.001; ^**^p < 0.01; ^*^p < 0.05; ns = not significant).

We observed that the density of Caspase 3-expressing apoptotic cells in the spinal cord 7 days after SCI/sham surgery was highest in the injured vehicle animals (264 ± 22 DAPI^+^&Caspase3^+^ cells/mm^2^) followed with distance by the injured NRA animals (153 ± 25 DAPI^+^&Caspase3^+^ cells/mm^2^). This difference was significant (*p* = 0.004). As expected, the density of apoptotic cells was significantly lower in uninjured Sham animals (14.2 ± 3.5 DAPI^+^ and Caspase3^+^ cells/mm^2^) compared to both, the vehicle, and the NRA group (*p* < 0.001, respectively; Figures 5A,B).

The immunointensity of chondroitin sulfate proteoglycans (CSPGs) in the spinal cord which serves as a marker for the extent of fibrous scar formation was significantly lower in the Sham group (23.5 ± 1.00) when compared to the NRA group (29.1 ± 0.98; *p* = 0.03) and the vehicle group (30.1 ± 1.67; *p* = 0.009) 7 days after SCI/sham surgery. However, there was no significant difference between the NRA-treated animals and the untreated animals in the vehicle group (*p* = 0.84; Figures 5C,D).

Similarly, we found that the immunointensity of GFAP, indicating spinal cord astrogliosis, was lowest in the uninjured Sham animals (18.9 ± 0.87) and showed a significant increase in the vehicle animals (41.0 ± 6.09; *p* = 0.006) as well as the NRA animals (40.3 ± 2.52; *p* = 0.007) at the end of the experiment. However, there was no significant difference between the NRA and the vehicle group (*p* > 0.99; Figures 5E,F).

### 3.6. Motor outcome

**Figure 6:**
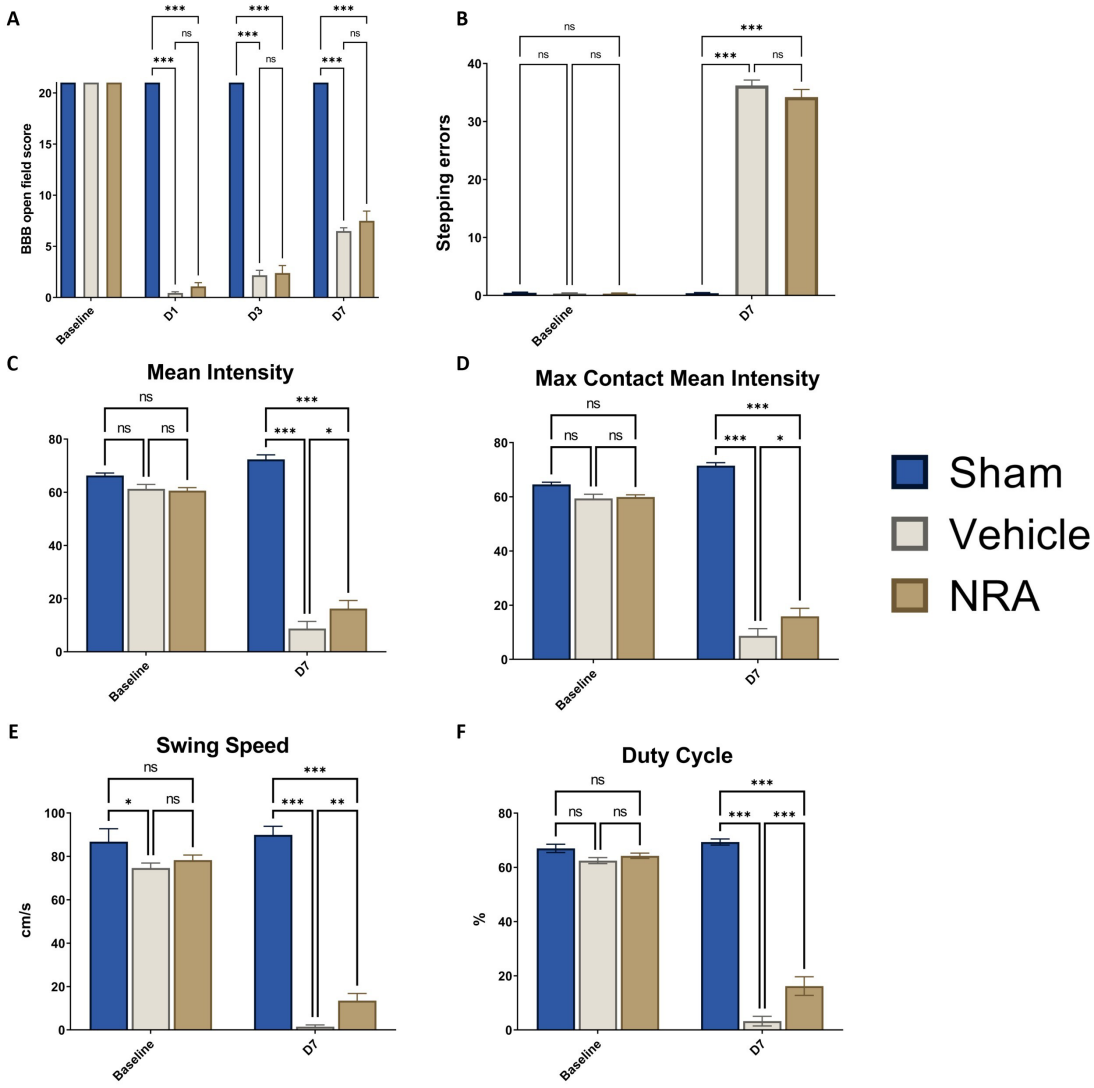
**(A)** Blood–brain–barrier (BBB) open field scores of the animals from baseline until 7 days after SCI/sham surgery. **(B)** Number of stepping errors in the Gridwalk test at baseline and 7 days after SCI/sham surgery. **(C)** Mean Intensity, **(D)** Max Contact Mean Intensity, **(E)** Swing Speed, and **(F)** duty cycle of the hindlimbs as CatWalk XT^®^ automated gait analysis parameters at baseline and 7 days after SCI/sham surgery (*n* = 12/group; two-way repeated measure ANOVAs, followed by Tukey tests for multiple comparisons; ^*^p < 0.05, ^**^p < 0.01, ^***^p < 0.001; ns = not significant).

To assess functional recovery of the hindlimbs, the BBB open field score was performed at baseline and over the course of the experiment. While all animals reached 21 points at baseline, the contusion/compression T9 SCI severely impacted on the hindlimb locomotion in the NRA and vehicle animals. One day after SCI, the BBB score dropped to 0.79 ± 0.26 points for the NRA group and to 0.44 ± 0.12 points for the vehicle group, with no statistically significant inter-group difference (*p* = 0.23), indicating almost no noticeable hindlimb movement and no early effect of the NRA-treatment. On day 3 after SCI, the BBB score gradually recovered to 2.39 ± 0.73 points for the NRA animals and to 2.18 ± 0.48 points for the vehicle animals, without reaching a significant difference either (*p* = 0.97). At the end of the experiment (day 7), the BBB score reached 7.50 ± 0.95 points in the NRA group and 6.50 ± 0.31 points in the vehicle group, indicating again only a limited and insignificant improvement of locomotion recovery with the 7-day-long i.t. NRA application (*p* = 0.59). In contrast, all Sham animals reached a BBB score of 21 at days 1, 3 and 7 after surgery (*p* < 0.001 for all timepoints; Figure 6A).

The Gridwalk test was performed at baseline and 7 days after SCI/sham surgery. While animals in the Sham group exhibited scarce stepping errors even at the end of the experiment (0.39 ± 0.12 at D7), the thoracic contusion/compression injury had a devastating effect that most of the animals in the NRA and vehicle group could only sweep their hindlimbs over the grid. As a result, 34.22 ± 1.31 stepping errors in the NRA animals and 36.22 ± 0.94 errors in the vehicle animals were counted 7 days after SCI without a statistically significant difference between both groups (*p* > 0.99; Figure 6B).

With the CatWalk XT^®^ gait analysis, we aimed to distinguish and amplify some of the earlier onset hindlimb gait parameters at baseline and 7 days after SCI/sham surgery, which is especially crucial for our study design.

Thereby, mean Intensity decreased vastly from baseline to 7 days after SCI in the NRA animals (60.62 ± 1.11 vs. 16.25 ± 3.04) and the vehicle animals (61.29 ± 1.65 vs. 8.77 ± 2.65; Figure 6C). Similarly, after the thoracic contusion/compression injury, Max Contact Mean Intensity dropped from baseline to 7 days after SCI in the NRA animals (59.91 ± 0.83 vs. 15.89 ± 2.95) and also the vehicle animals (59.42 ± 1.51 vs. 8.73 ± 2.63; Figure 6D). Nevertheless, at the end of the experiment, the 7-day-long NRA-treatment had led to a significantly better recovery in terms of Mean Intensity (*p* = 0.04) and Max Contact mean intensity (*p* = 0.04) when compared to uninjured vehicle animals. As expected, Sham animals performed significantly better than injured NRA and vehicle animals in both parameters (*p* < 0.001 each; Figures 6C,D).

Swing Speed was found to be significantly faster in the NRA group than the vehicle group 7 days after SCI (13.50 ± 3.32 cm/s vs. 1.52 ± 0.79 cm/s, *p* = 0.005; Figure 6E), while Sham animals performed significantly better than both injured groups (89.94 ± 3.90 cm/s; *p* < 0.001, respectively).

Lastly, although the Duty Cycle of the NRA animals declined dramatically from baseline to 7 days after SCI (64.27 ± 0.98% vs. 16.19 ± 3.44%) when compared to the vehicle animals (3.25 ± 1.78%), it still displayed a significantly longer stepping period (*p* < 0.001). Similarly, animals in the Sham group (69.36 ± 1.13%) also performed significantly better than the NRA and vehicle group at the end of the experiment (*p* < 0.001 each; Figure 6F).

## 4. Discussion

In our current study, we have used the lipid-soluble NK1R antagonist EUC-001 as a treatment for experimental SCI in rats. Moreover, in contrast to previous reports, we have administered this drug continuously and topically (intrathecally) to the severely injured spinal cord for up to 7 days.

As a lipid-soluble drug, EUC-001 has several potential benefits compared to water-soluble NRAs such as NAT: It is well recognized that there is a rapid disruption of the BSCB function following SCI. However, the rate and nature of barrier re-establishment are more challenging to determine, although it does correlate with improved function (Bartanusz et al., 2011). In relation to the BBB, NRAs can play an active role in rapidly restoring BBB function following traumatic injury (Donkin et al., 2009). If an NRA produces the same effect in relation to the BSCB, in other words, ameliorating barrier dysfunction, this may impede the subsequent diffusion of the antagonist to the injury site, if that antagonist has a low lipid solubility. Hence, a more lipid soluble agent may be beneficial in relation to a prolonged administration regimen. Another factor to take into consideration is the hypoperfusion occurring at the injury site which makes it more difficult for water-soluble agents to reach their target site (Couillard-Despres et al., 2017). In contrast, by local administration of a lipid-soluble agent to the spinal cord, the injury site may be reached by simple diffusion, facilitating the drugs effect. For EUC-001, this could be performed during, e.g., an emergency decompression surgery, rendering it a potential candidate for clinical translation.

Before assessing potential treatment effects of EUC-001, we aimed to confirm its antagonizing capacity on SP and NK-1R expression: In accordance with Leonard et al. (2013), we observed a similar pattern of the anatomical distribution of SP and NK-1R in the spinal cord’s dorsal horn or around the lesion site. In addition, the NK-1R expression was predominantly spotted in the white matter, where the density of DAPI^+^&NK-1R^+^ cells was significantly reduced after NRA treatment. More importantly, SP and the NK-1R were significantly decreased after the 7-day-long, intrathecal EUC-001 treatment. These findings allowed us to further investigate the downstream effects generated by effectively blocking the neurogenic inflammation through a neurokinin-involved pathway.

Because of previously observed positive effects of NK1R-antagonization on tissue edema formation (Nimmo et al., 2004), we next used the wet & dry weight analysis and MR imaging to assess the severity of spinal cord edema after SCI with or without EUC-001 treatment. Such effects of the NRA-treatment would be desirable because posttraumatic spinal cord edema provokes a series of consequences, including reduced perfusion, the release of neurotoxic substances and free radicals, necrosis, and apoptosis, resulting in progressive inflammation and, thus, motor dysfunction in patients (Wagner et al., 2012).

Although the wet and dry weight analysis is a well-recognized measurement to evaluate tissue edema (Li et al., 2019; Chen et al., 2020; Gattarello et al., 2021), and despite our efforts to minimize potential bias by correcting the measurements of the injured thoracic spinal cord with the intact cervical spinal cord for every animal, we could not observe a significantly increased spinal cord edema between the uninjured Sham and the SCI animals. Correspondingly, the NRA-treatment led to no relevant reduction of tissue water 7 days after SCI. Of note, since we used 10 mm-long spinal cord pieces to assess the wet & dry weight, “uninjured” tissue surrounding the lesion epicenter might have influenced the edema measurement and weakened this methodology. Meanwhile, in a study by Leonard et al. (2014b), the non-lipid-soluble NK1R-antagonist n-acetyl L-tryptophan, given intravenously to rabbits as three bolus injections after a thoracic balloon compression SCI, did not result in a significant reduction of the tissue water content in the wet & dry analysis either, despite larger animals being used. However, the authors observed a significant increase of spinal cord edema compared to uninjured rabbits 3 days after SCI which contrasts with our findings in rats 7 days after the injury. Indeed, contradicting outcomes on spinal cord edema have been reported with the wet and dry weight analysis after clip compression SCI before (Li and Tator, 1999; Jin et al., 2014).

Thus, compared to this method, measuring the signal intensity of T2—weighted MR images as a surrogate for tissue water content might be more accurate in rodent SCI models, and moreover is the primary clinical technique to visualize spinal cord compression and edema in SCI patients. Nout et al. (2009), monitored the MRI indices of swelling, edema, and hemorrhage within the spinal cord after a hypertonic saline administration to mimic secondary SCI in rats, and their result demonstrated a high spatial and temporal correlation between lesion progression and MRI signal changes. Similar findings have been reported by Yang et al. (2022) in rabbits. In clinical practice, the Brain and Spinal Injury Center (BASIC) score, a classification system for grading spinal, cord hemorrhage and edema based on the axial extent of intramedullary signal abnormality on T2 weighted MRI, has been recommended to evaluate the prognosis guide decision making the first day after admission (Freund et al., 2019). In previous reports on NK1R-antagonization after experimental SCI, MRI has not yet been implemented to assess spinal cord edema. Using a 1 T MR scanner, we observed a significant increase of spinal cord edema 7 days after SCI in the vehicle group compared to the injured Sham group. However, the effect of the NRA administration was limited at all measured time points. These findings might be related to the fact, that it is more challenging to acquire high-resolution images in animals than in humans, which makes it almost impossible to use established scoring systems like the brain and spinal injury center (BASIC) score, which has been proven to have substantial diagnostic and prognostic value in SCI patients (Talbott et al., 2015). Nevertheless, MR scanners with higher field strengths or techniques such as diffusion-weighted imaging (DWI), used to measure the diffusion of water in tissue, are already available in the clinical setting and might be able to infer more information about the microstructure of the damaged spinal cord in rodent SCI models in the future (Wilkins et al., 2020).

Spinal cord edema after SCI is closely related to the posttraumatic disruption of the BSCB, which comprises adherents/tight junctional protein complexes, astrocytic endfeet, microglia, pericytes, and endothelial cells and protect the CNS from neurotoxic substances and molecules from the peripheral circulation (Bartanusz et al., 2011; Xanthos et al., 2012). The BSCB is furthermore crucial for the pathophysiological regulation and interaction between the CNS and the peripheral immune system. While different techniques to assess the BSCB integrity exist, we based our analyses on surrogates such as the extravasation of Fibrinogen, the infiltration of T-Lymphocytes and the presence of CD31^+^ endothelial cells in the injured spinal cord. Fibrinogen, which circulates throughout the blood vessels, is a glycoprotein synthesized by the liver and connected by disulfide bonds. In the CNS, Fibrinogen is unique among plasma proteins owing that it contains binding sites for receptors expressed by CNS cells and proteins that mediate key CNS pathophysiological processes (Petersen et al., 2018). Moreover, the half-life of SP can be prolonged by forming a complex with high molecular weight factors such as Fibrinogen (Suvas, 2017). In our study, we found that the presence of Fibrinogen and thus its extravasation over the BSCB was significantly increased in the injured spinal cord after 7 days compared to the intact spinal cord in Sham animals, indicating a posttraumatic disruption of the BSCB. Importantly, this disruption was reduced by the treatment with EUC-001, characterized by significantly less Fibrinogen extravasation, suggesting a positive effect of the drug on the maintenance of the BSCB. Similarly, we found that the thoracic contusion/compression SCI had led to a significantly increased cell density of T-lymphocytes after 7 days. Whether this increase of T-lymphocytes after SCI is related to a reduced BSCB permeability or neurogenic inflammation is still a matter of debate. However, SP release led to a dose-dependent increase in T lymphocyte adherence in other studies, implying facilitation of peripheral immune T cell infiltration into the CNS, other than a local recruiting mechanism (Corrigan et al., 2016; Zhang et al., 2021). Nevertheless, in our study, the continuous application of EUC-001 was able to significantly reduce the T-lymphocytic infiltration. Endothelial cells, characterized by the expression of CD31, constitute the inner lining of blood vessel in the spinal cord. Specifically, CD31, also known as platelet endothelial cell adhesion molecule (PECAM-1) plays an important role in paracellular permeability, cellular adhesion, and leukocyte diapedesis (Privratsky and Newman, 2014; Morris et al., 2020). We, therefore, also quantified the presence of such endothelial cells in the spinal cord 7 days after SCI/sham surgery and noticed a significant increase in the injured vehicle group compared to the uninjured Sham group, but not in the NRA group. These findings highlight a cellular alteration of the BSCB after injury, suggesting higher permeability for leukocytes which could only be slightly improved by the treatment with EUC-001 (Mao et al., 2020). Taken together, intrathecal administration of the cell-permeable EUC-might have contributed to strengthen the integrity of the BSCB after SCI in rats which contrasts with findings of Leonard et al. (2014b), who could not observe such effects of the NK1R-antagonization in rabbits. Of note, this difference might be attributed to the fact, that they used a different species, but more importantly that they administered an NK1R antagonist intravenously and only as bolus injections, which has been critically discussed by the authors themselves.

Microglia act as immune cells in the CNS and are activated and mobilized after SCI in response to, e.g., the secretion of neurotoxins and proinflammatory cytokines, but also to the binding of SP and NK1R (Orr and Gensel, 2018). Thus, as a measure of posttraumatic neuroinflammation, we quantified the density of infiltrating macrophages and activated resident microglia in the spinal cord, characterized by the colocalization of their respective markers Iba1 and TMEM119 with DAPI (González Ibanez et al., 2019). Hereby, we discovered a significantly enhanced infiltration of macrophages and activation of resident microglia 7 days after the injury. However, the modulating effect of EUC-001 on these aspects of neuroinflammation was very limited. To provide a more detailed assessment of neuroinflammation, we further evaluated the polarization of the spinal cord microglia population into M2-subtype which has anti-inflammatory properties and is beneficial to neuroregeneration (Han et al., 2018). By quantifying the specific M2-microglial marker Arginase 1, we found that EUC-001 has no effect on the anti-inflammatory microglia polarization as early as 7 days after the injury. Only Leonard et al. (2014b) also investigated microglial immunoreactivity in the spinal cord after SCI with and without the systemic application of the NK1R-antagonist n-acetyl L-tryptophan. In their study, they could not demonstrate a significant attenuation of microglial activation up to 14 days after the drug treatment in rabbits. Given the shorter observation time in our current experiment, it remains unclear whether EUC-001 would be able to positively affect microglia-related neuroinflammation in the longer term, which should be assessed in future studies.

The loss of cells in the injured spinal cord by, e.g., apoptosis is another hallmark of the secondary injury cascade, leading to persisting neurological deficits and functional recovery (Jiang et al., 2022). The number of apoptotic cells can conversely serve as a surrogate for the number of spared tissue and thus the potential capacity for neuroregeneration after SCI. With the EUC-001 treatment, we were indeed able to markedly reduce apoptosis 7 days after the injury, which could provide a basis for improved functional recovery.

Nevertheless, other aspects of tissue morphology in the injured spinal cord remained unaffected even by the EUC-001 treatment: The accumulation of chondroitin sulfate proteoglycans (CSPGs) in the extracellular matrix of the spinal cord contributes to the fibrous scar formation after SCI and inhibits axonal growth and reconnection (Siebert and Osterhout, 2011). By measuring the immunointensity of a CSPG staining, we found that tissue scarring in the spinal cord was already significantly increased 7 days after SCI but that there was no measurable difference between NRA and vehicle animals. In the context of SCI, reactive astrocytes are regarded to be harmful because they predominantly participate in the formation of glial scars and posttraumatic cystic cavities, which hinder axonal growth and neuronal plasticity, especially in the chronic postinjury phase (Younsi et al., 2021). By analyzing the immunointensity of GFAP, we again observed a significant effect of the injury already after 7 days with increased astrogliosis in both, the NRA, and the vehicle group. However, the difference in astrogliosis caused by EUC-001 administration was negligible. Therefore, we hypothesized that the treatment with EUC-001 alone might not be sufficient to significantly tissue scarring and astrogliosis, which are modulated by multiple mechanisms, or that the effect simply does not manifest in the subacute but rather in the chronic postinjury stage.

Ultimately, all efforts to attenuate harmful secondary injury processes with drugs such as EUC-001 have the same goal of improving functional recovery. For rodent SCI models, the BBB open field score is mostly used to assess the hindlimb motor function because it allows the evaluation of all levels of locomotion deficits, including joint movement, weight-bearing, coordination, and trunk stability (Basso et al., 1995). Although the NRA animals in our study had the highest BBB scores 7 days after SCI, not even they reached the 9 points required for weight-bearing. Moreover, no relevant effect of the drug treatment could be observed. Accordingly, as a consequence of the severe contusion/compression SCI applied to the thoracic spinal cord, many injured animals only presented retraction or sweeping over the floor as their hindlimb joint movement and, therefore, had a high number of missteps in the Gridwalk test. This behavioral assessment is supposed to measure the status and change of fine sensory function and motor movement, as well as general coordination, which were not improved by EUC-001 application in our experiment. Of note, due to the severity of the injury, the BBB score and the Gridwalk test might not have a sufficiently high sensitivity to discern positive effects of the drug treatment. Thus, we additionally implemented the CatWalk XT® gait analysis, which allows the sensitive and objective generation of over 100 gait parameters, from which suitable ones can be chosen to evaluate functional recovery after rodent SCI according to the selected injury model (Hamers et al., 2001; Walter et al., 2020; Zheng et al., 2023). For the thoracic contusion/compression injury, static CatWalk XT^®^ gait analysis parameters (paw intensity measurements) or preliminary locomotive parameters (swing speed and duty cycle) are more suitable than parameters focusing on weight bearing (base of support), general locomotion (average speed and stride length), or coordination (regularity index). By choosing the parameters Mean Intensity and Max Contact Mean Intensity we were able to show that the pressure of a complete paw (during the entire run or the maximum contact) is significantly improved with the NRA-treatment 7 days after SCI, indicating increased muscle tone of the hindlimbs. Similarly, the parameter Swing Speed which denotes the speed of the paws during a swing, indicated significantly improved muscle tone and locomotion with the drug treatment. And moreover, the parameter Duty Cycle, which expresses the stand as a percentage of the period between two consecutive initial contacts of the same paw and mirrors the general locomotion and strength of the hindlimbs was significantly increased after application of EUC-001 as well. Interestingly, Leonard et al. (2014b) found a significant improvement of the motor outcome in rabbits using a modified Tarlov score exactly 6 days after SCI with their i.v. n-acetyl L-tryptophan treatment, which corresponds to our CatWalk XT^®^ gait analysis results in rats. However, this improvement was not detectable anymore at later timepoints, raising the question whether the bolus administration was sufficient to induce prolonged effects of the drug treatment or whether rabbits as a species reacted. Taken together, by using the CatWalk XT^®^ gait analysis, we were able to highlight some distinctive features of an early onset locomotion recovery mediated by the antagonization of the NK1R, which potentially did not translate to the more global assessments Gridwalk Test and BBB open field score yet.

However, our study lacks further functional measurements which is a limitation for the comprehensive assessment of EUC-001. Other authors have used the prick test to evaluate the recovery of sensory function or cystometry and external urethral sphincter (EUS) EMG to measure the spinal micturition reflex and bladder voiding after SCI (Zhang et al., 2008; Leonard et al., 2014b). Interestingly, while no improved recovery of sensory function could be observed after the i.v. administration of n-acetyl L-tryptophan in a thoracic compression SCI model, the i.t. administration of another non-lipid-soluble NK1R-antagonist, L-733060, led to blockage of the spinal micturition reflex and phasic/nonphasic voiding contractions after a thoracic transection SCI. Such findings suggest that SP and its receptors might play a relevant role in sensory function and more specifically the spinal micturition as well, which should be taken into consideration when planning similar experiments.

Further limitations of our study must be noted: Firstly, we did not assess histological regeneration or behavioral recovery beyond 1 week after SCI, which would further have elucidated the effects of EUC-001 in the subacute or chronic phase post-injury phase and should be performed in future experiments. Secondly, it remains unclear whether the 5-day-long administration of analgesics after SCI (which was an animal ethics requirement) could have affected the behavioral assessment or even the level of SP-related inflammation or neuropathic pain itself. Although all animals received the same analgesic regimen and although all behavioral tests were always performed before the daily analgesic administration, we still cannot rule out a potential bias. Lastly, since some pharmacokinetic reports suggest a 10:1 intravenous to intrathecal conversion ratio for lipid-soluble drugs, the i.t. dose of EUC-001 we administered (10 mg/kg) might have been relatively high and should be reconsidered for human trials (Gorlin et al., 2016).

In conclusion, with our current study, we were able to demonstrate that the 7-day-long intrathecal administration of EUC-001, a cell-permeable and lipid-soluble NK1R-antagonist, leads to support of the BSCB in terms of immune cell and protein infiltration, reduction of apoptosis, and distinct early hindlimb motor recovery 7 days after a severe thoracic contusion/compression SCI in rats. However, its beneficial effect on reducing spinal cord edema, microglia activation, tissue scarring, astrogliosis, and general locomotion recovery remains limited. Nevertheless, compared to previous reports on non-lipid-soluble NK1R-antagonists, EUC-001 might have a better efficacy profile in the context of SCI and should be studied further.

## Data availability statement

The raw data supporting the conclusions of this article will be made available by the authors, without undue reservation.

## Ethics statement

The animal study was reviewed and approved by Animal Care Committee of the federal government of Baden Württemberg, Germany (ethic approval code G-285/19).

## Author contributions

GZ and AY: conceptualization. GZ, A-KH, AN, KZ, and AY: methodology. GZ, A-KH, MT, and HZ: acquiring data. GZ and A-KH: formal analysis. AN, TS, KK, and AU: providing reagents. GZ, A-KH, and AY: writing. All authors have read and agreed to the published version of the manuscript.

## Conflict of interest

The authors declare that the research was conducted in the absence of any commercial or financial relationships that could be construed as a potential conflict of interest.

## Publisher’s note

All claims expressed in this article are solely those of the authors and do not necessarily represent those of their affiliated organizations, or those of the publisher, the editors and the reviewers. Any product that may be evaluated in this article, or claim that may be made by its manufacturer, is not guaranteed or endorsed by the publisher.
